# Prevalence and Associated Factors of Common Mental Disorders Among Pregnant Mothers in Rural Eastern Ethiopia

**DOI:** 10.3389/fpsyt.2022.843984

**Published:** 2022-03-28

**Authors:** Dawit Tamiru, Tadesse Misgana, Mandaras Tariku, Dejene Tesfaye, Daniel Alemu, Adisu Birhanu Weldesenbet, Berhe Gebremichael, Merga Dheresa

**Affiliations:** ^1^Department of Midwifery, College of Health and Medical Sciences, Haramaya University, Harar, Ethiopia; ^2^Department of Psychiatry, College of Health and Medical Sciences, Haramaya University, Harar, Ethiopia; ^3^School of Public Health, College of Health and Medical Sciences, Haramaya University, Harar, Ethiopia; ^4^School of Nursing and Midwifery, College of Health and Medical Sciences, Haramaya University, Harar, Ethiopia

**Keywords:** common mental disorder, pregnancy, antenatal care, gestational age, rural Ethiopia

## Abstract

**Background:**

Antenatal common mental disorder is a significant public health issue, especially in low- and middle-income countries with an extensive treatment gap. Common mental disorders have multifaceted implications on maternal and fetal health outcomes during pregnancy with long-running economic and social sequels. This study aimed to determine the prevalence of common mental disorder and associated factors among pregnant mothers in eastern Ethiopia, Kersa and Haramaya Health, and Demographic surveillance sites.

**Methods:**

A community-based cross-sectional study was conducted in Kersa and Haramaya health and demographic surveillance sites from January 30 to April 30, 2021. World Health Organization Self-Reporting Questionnaire (SRQ-20) was used to measure common mental disorder among 1,015 randomly selected pregnant women. Data were collected face-to-face using open data kit software. Logistic regression was fitted to identify factors associated with common mental disorders.

**Results:**

The overall prevalence of common mental disorders (SRQ > 6) among pregnant women was 37.5% (95% CI: 34.5, 40.5). Current substance use (AOR = 1.99, 95% CI 1.37, 2.88), intimate partner violence (AOR = 2.67, 95% CI 2.02, 3.53), null parity (AOR = 3.10, 95% CI 1.65, 5.84), gestational age [first trimester (AOR = 2.22, 95% CI 1.01, 4.93) and third trimester (AOR = 1.74, 95% CI 1.31, 2.31)], history of abortion (AOR = 2.03, 95% CI 1.27, 3.24), and absence of antenatal care follow-up (AOR = 1.43, 95% CI 1.08, 1.89) were significantly associated with common mental disorder during pregnancy.

**Conclusion:**

Common mental disorders are prevalent among pregnant women in the study area with significant correlates. Administration of regular screening programs for maternal mental health conditions in rural, low-income communities, integrating into primary health care settings is imperative to reduce the risk.

## Introduction

Globally more than one billion people are affected by mental or addictive disorders, causing 19% of all years lived with disability (YLD) (1). Common mental disorders (CMDs) refer to a range of non-psychotic mental health conditions such as anxiety, depressive and somatoform disorders (1–3). The conditions are rampant in the population hence why they are deemed common and impact the mood or feelings of affected person. It is also common people experiencing more of these conditions simultaneously (3, 4).

The number of individuals with a CMD is increasing globally, mainly in lower-income countries, because of the increment in population and more people living to the age when depression and anxiety are common (2, 5). Globally, it is estimated that 3.6% (264 million) and 4.4% (322 million) and of the world’s population suffer from anxiety and depressive disorders, respectively. Estimates vary across regions, from a low of 2.6% in males in the western Pacific region to 7.7% among females in the Americas. In Ethiopia, 3.3% of the population suffers from anxiety disorders, while 4.7% face depression causing 3.5 and 10.1% of total YLD, respectively (2). The disorders are more common among females than males, particularly in pregnant women (2, 5, 6).

Mental health in pregnant women is a significant public health matter, especially in low- and middle-income countries (LMICs) (7, 8). Although pregnancy is generally considered a period of contentment, the state increases vulnerability to psychiatric conditions like common mental disorders (9). Even though symptom scores for depression in studies are elevated in antepartum compared to after childbirth, postnatal depression has been a focus of concern, while depression during pregnancy has been relatively neglected (10). The prevalence of CMDs among pregnant women ranges from 1 to 37%, with an increasing threshold, mainly in low and middle-income countries (11–13). However, common mental disorders during pregnancy are often left under diagnosed and untreated because conditions are usually ascribed to typical experiences of pregnancy (14).

Untreated CMD during pregnancy can lead to the negative child, mother, and family conditions, including poor antenatal clinic follow-up, poor nutrition and self-care, higher substance abuse, suicidal ideation, and thoughts of hurting the fetus, development of depression after childbirth, neglect of the child, and family breakdown (15, 16). CMDs during the antenatal period have also been associated with adverse pregnancy and child well-being effects, including lower birth weight, prematurity, neonatal mortality, and deficient child nutrition status, behavioral, emotional, and cognitive development (17, 18).

Multiple constituents and psychosocial determinants have been distinguished as contributing to common mental disorders during pregnancy. The most common ascertained circumstances include lack of social support, history of violence, pressure to have a child, history of mental illness, unplanned pregnancy, and adverse life events, complications in past or index pregnancy, and pregnancy loss (13, 19, 20).

In LMICs, the treatment gap for mental health conditions is estimated to be over 80% (21). The high mental health burden among pregnant women accentuates the need for mental health services within the maternal health care system in low-income settings (22). The World Health Organization (23) endorsed maternal mental health care be integrated into primary health care for improved access (24, 25). Integration of CMD screening with antenatal care has also been mentioned as an effective strategy to realize WHO’s Mental Health Gap Action Program (MhGAP), which will lead to better maternal and child health outcomes (23, 26). Although Ethiopia has recently integrated mental health services into the primary healthcare system, the implementation faces many barriers (27, 28).

Ethiopia is galvanizing efforts to achieve continuum Growth and Transformation Plan II (GTP II) and Sustainable Development Goals (SDG). The need for interdisciplinary and inter-sectorial solutions to reach maternal and child health, as well as mental health goals, is highlighted (29).

Attitudes and professional treatment-seeking behavior for mental health problems in Ethiopia are generally low; doubt of discrimination for having a mental disorder, depending in non-formal aid source, and negative beliefs toward mental health services were the most commonly cited barriers (30, 31). Despite effective treatments are available, studies reported that many affected people do not seek professional help. It was indicated that less than 35% of adults with identifiable mental problems looked for professional help, in which more than 80% visited informal help sources (31, 32). Modern mental health services centers are concentrated in urban settings, which are not accessible to the majority of the population.

Describing the correlates of common mental disorders in pregnancy and childbirth is a crucial step in preventing and treating of the associated complications in lower-income countries. With a largely rural population, low mental health care service, and high maternal, infant, and neonatal mortality rates, Ethiopia lacks reliable data on the burden and effects of mental disorders (33, 34). Demographic and health surveys in the country generally have not included maternal mental health status as expected health outcomes and national data are rarely available; thus, the exact extent of the broader impact remains unclear and likely under-estimated (34, 35).

When resources are available, data are predominantly from health facility surveys, whereas a high proportion of pregnant women remain at home without seeking maternal health care service (34, 36). Therefore, this study aimed to determine the magnitude and determinants of common mental disorders during pregnancy in the eastern region of Ethiopia.

## Materials and Methods

### Study Setting, Design, and Period

A community-based cross-sectional study was conducted in Kersa and Haramaya Health and Demographic Surveillance Sites (HDSS) from January 30 to April 30, 2021.

Kersa and Haramaya Health and Demographic Surveillance Site is one of the seven HDSS sites in the Ethiopian universities intended to reflect the countries’ health and demography. Kersa and Haramaya Demographic Surveillance and Health Research Center (KDS-HRC) was established in 2007. Kersa HDSS is located in Kersa District, Oromia Regional State, eastern Ethiopia. There are 35 rural sub-districts (Kebeles) and three small-town kebeles. The Kersa HDSS covers 24 of the 38 kebeles. Most inhabitants are farmers, with a small numbers working in trade, public service, or casual laborers.

Haramaya Woreda (district) is located in the East Hararghe Zone of Oromia Regional State. Haramaya Woreda has thirty-three rural and two urban kebeles (sub-districts). Haramaya HDSS covers 12 rural kebeles.

Rural eastern Ethiopia is where the majority of Khat (*Catha edulis*), psychoactive substances, in the country produced and exported abroad (37).

### Study Population

All pregnant women living in Kersa and Haramaya Health and Demographic Surveillance Sites (HDSS) were the source population. All registered pregnant women were enrolled in the study irrespective of their age and pregnancy trimester. Pregnant women were located from Health and Demographic surveillance sites registration server and interviewed in their homes. Pregnant women who could not communicate due to serious medical and psychiatric illnesses were excluded from the study.

### Sample Size Determination

The minimum sample size required for this study was determined using a single population proportion formula with assumptions of confidence level at 95% = 1.96, a margin of error (d) = 0.04, and a reasonable proportion of common mental disorder (*P* = 0.267) (38). After incorporating a design effect of 2 and a 10% non-response rate, the maximum sample size calculated for this study was 1,034. The calculated sample size was proportionally allocated based on the confirmed number of pregnant women in selected Kebeles (clusters); the study participants were then determined using simple random sampling.

### Study Variables and Measurements

Data was collected using Open Data Kit (ODK) tools. Observations were uploaded to the ODK aggregate server. The data collection instrument included socio-demographic and economic characteristics, reproductive, obstetric and gynecologic conditions, pre-existing medical and psychiatric conditions, substance use, intimate partner violence, and common mental disorder.

Self-Reporting Questionnaire (SRQ-20) was used to measure common antenatal mental disorders. The SRQ-20 is composed of twenty yes/no items asking about depressive, anxiety, panic, and somatic symptoms during the preceding 30 days. For this study, the total score was dichotomized (SRQ-20 < 6 versus SRQ ≥ 6), with high scores indicating a high level of CMD (39). Data on household economic status were collected using a tool adapted and modified from the Ethiopian Demographic and Health Survey (EDHS) of 2016 (40). Alcohol, Smoking, and Substance Involvement Screening Test (41) comprising eight questions or items covering ten substances, was used assessing substance use behavior of pregnant women. Khat was included under the stimulant category, and shisha was incorporated under tobacco. The experience of intimate partner violence (IPV) was assessed using the WHO multi-country study questionnaire constituting psychological, physical, and sexually violent acts, often accompanied by controlling behavior where a single positive answer indicated the presence of violence (42).

### Data Collectors and Data Collection Procedure

Twenty data collectors who have at least a B.Sc. degree in health sciences and working in Kersa and Haramaya Demographic Surveillance and Health Research Center (KDS-HRC) collected the data for this study. Besides, they were supervised by six M.Sc. holders. In addition, an intensive 3 days training was provided to data collectors and supervisors.

The data were collected by face-to-face interview using structured and semi-structured questionnaires prepared in ODK collect form, completed data were sent directly to the server. Pregnant women were interviewed in separate rooms in their home environment or inside their compounds during working hours 7 days of the week.

### Data Quality Control

To maintain the consistency of the data collection tool, the questionnaire was prepared in English language and translated to local languages, Afaan Oromo and Amharic languages, and vice versa. To evaluate the acceptability and applicability of the procedures and tools, pre-testing was done on 5% of the sample size 1 week before the actual data collection in the kebeles that are not included in the samples from each site. To keep the completeness and consistency of the questionnaire, data collectors were closely supervised during the data collection process by the supervisors and investigators.

### Data Management and Analysis

After data files were downloaded from the server, the data set were exported to Stata version 14.0 for cleaning, coding, and analysis. Categorical variables were described using simple frequencies and percentages. Continuous variables were defined using mean or median and inter-quartile ranges depending on their distribution and standard deviation. Bivariate analysis was done using binary logistic regression to see the association between each independent variable and common mental disorder. The final multivariate analysis model included all variables from the bivariate analysis with *p* ≤ 0.2 to control all possible confounders. The direction and strength of statistical association were measured by odds ratio with 95% CI. Finally, a *P*-value less than 0.05 were considered to declare the presence of a statistically significant association. The Hosmer–Lemeshow statistic and Omnibus test tested the model goodness of fit. The multi co-linearity test was also carried out to see the correlation between independent variables using VIF, standard error (SE), and tolerance tests.

### Ethics Approval and Consent to Participate

Ethical approval was obtained from Haramaya University, College of Health and Medical Sciences, Institutional Health Research Ethics Review Committee (IHRERC) and submitted to the respective health bureau of each study site. The study was conducted under the Declaration of Helsinki and, participants were informed that participation in the study is voluntary; had the right to withdraw or refuse to participate in the study at any time. Throughout the study period, the confidentiality of the data was strictly followed; participants were interviewed in separate rooms in their home environment. Before starting data collection, information sheets are read, and written and signed consent was obtained from each participant. Pregnant women who screened positive for the common mental disorder were linked to health facilities for assessment and interventions.

## Results

### Socio-Demographic Characteristics

From a total of 1,034 participants selected for the study, 1,015 consented to participate in the study yielding a response rate of 98.2%. Among the participants, 676 (66.6%) were between the age of 20–35 years with a mean of 30.1 years (SD = 8.5). The majority of the study participants, 982 (96.75%) were married in their current relationship, 1,012 (99.7%) were Muslim followers, and 1,010 (99.51%) were Oromo in ethnicity. About 772 (76.06%) of the participants neither read nor write and 900 (88.67) were housewives. About 388 (38.2%) of the participants were on a lesser quintile of wealth index (Table 1).

**TABLE 1:** Socio-demographic and economic characteristics of pregnant women.

Variables	Category	Frequency	Percentage (%)
Age	<20	94	9.26
	20–35	676	66.60
	>35	245	24.14
Marital status	Married	982	96.75
	Others^a^	33	3.25
Religion	Muslim	1,012	99.70
	Orthodox	3	0.30
Ethnicity	Oromo	1,010	99.51
	Amhara	5	0.49
Educational status	High school and above	218	21.48
	Can read and write	25	2.46
	Neither read nor write	772	76.06
Occupation	Farmer	47	4.63
	Housewife	900	88.67
	Others^b^	68	6.70
Wealth index	First quintile	388	38.23
	Second quintile	375	36.95
	Third quintile	252	24.83

*^a^Single, divorced, and widowed.*

*^b^Petty trader, student, retired, and unemployed.*

### Clinical, Reproductive, Obstetric, and Gynecological Characteristics

Among the study participants, 26 (2.56%) had a previous history of mental illness with a mean duration of 2.7 years (SD = 1.37). Of them, 10 (38.46%) were on the treatment. Twenty-three (2.27%) had a chronic medical illness, the majority (43.5%) being hypertension.

The majority of the study participants were grand multipara, woman who has had ≥5 births (live or stillborn) ≥28 weeks of gestation, (46.50%). Over half, 52.12% of the participants were in the third trimester and the mean gestational age of pregnant mothers was 28.91 (SD = 6.63) weeks. About 98 (9.66%) of the participants had a previous history of abortion while 48.08% have Antenatal Care (ANC) follow-up. About 70 (6.9%) of the participants had a history of a gynecological problem, of this tumor (41.43%) was the most prevalent one. The median age of pregnancy interval and marriage was 1.7 years (IQR = 0.56) and 17.7 years (IQR = 2.12), respectively (Table 2).

**TABLE 2:** Medical, reproductive, obstetric, and gynecological characteristics of pregnant women.

Variables	Category	Frequency	Percentage (%)
History of mental illness	Yes	26	2.56
	No	989	97.44
History of chronic medical illness	Yes	23	2.27
	No	992	97.73
Parity	Nulliparous	52	5.12
	Multipara (has had two or more births ≥28 weeks of gestation)	491	48.37
	Grand multipara (has had ≥5 births ≥28 weeks of gestation)	472	46.50
Gestational age (in a week)	First trimester	31	3.05
	Second trimester	455	44.83
	Third trimester	529	52.12
History of abortion	Yes	98	9.66
	No	917	90.34
ANC follow up	Yes	488	48.08
	No	527	51.92
Interval between last delivery and LNMP	<24 months	754	92.29
	24–59	55	6.73
	>59	8	0.98
History of gynecological problem	Yes	70	6.90
	No	945	93.10
History of gynecological operation	Yes	46	4.53
	No	969	95.47

### Psychosocial and Substance Use Behavior of Participants

**FIGURE 1:**
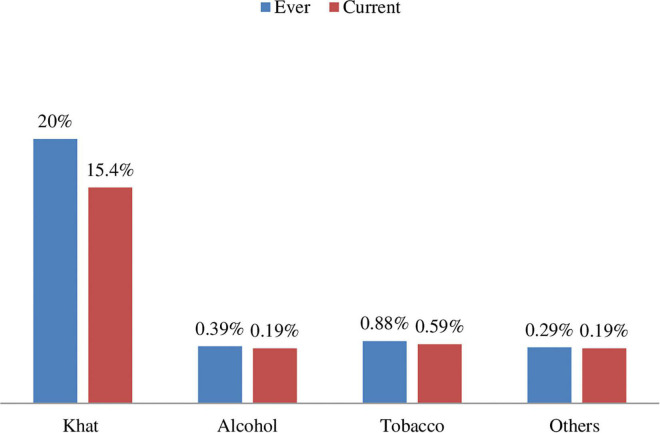
Description of the types of substance used by pregnant mothers.

Based on the assessment made on substance abuse using ASSIST, about 207 (20.39%) and 158 (15.57%) of the participants reported as they were used any type of substance at least once in their lifetime and the last 3 months, respectively (Figure 1). Concerning psycho-social factors, 493 (48.57%) of women reported that they experienced any act of physical, sexual, or emotional (psychological) violence or any combination of the three by an intimate partner during their current pregnancy.

### Prevalence of Common Mental Disorders

The overall prevalence of common mental disorder (SRQ > 6) among pregnant mother was found to be 37.5% (95% CI: 34.5, 40.5). About 180 (17.73%) of the women had no CMD symptoms and 454 (44.73%) had low CMD symptoms. Regarding the distribution of the symptoms, poor appetite (*n* = 350) was the most complained symptom followed by sleep disturbance (*n* = 285). The median SRQ-20 score was 2 (25th centile 1, 75th centile 8).

### Factors Associated With Common Mental Disorders Among Pregnant Mother

On bivariate analysis, age, wealth index, history of mental illness, history of chronic medical illness, current substance use, intimate partner violence, parity, gestational age, history of abortion, ANC follow up, history of the gynecological problem and gynecological operation had *p*-value score of less than 0.2 and entered into the multivariable analysis. In the multivariable logistic regression analysis model, however, only current substance use, intimate partner violence, parity, gestational age, history of abortion, and ANC follow-up were significantly associated with common mental disorders among pregnant women at a *p*-value of <0.05.

Pregnant women who use any type of substance in the past 3 months were two times (AOR = 1.99, 95% CI 1.37, 2.88) more likely to have CMD as compared to those women who didn’t use any types of substance. Those pregnant women who reported that they experienced intimate partner violence were 2.67 times (AOR = 2.67, 95% CI 2.02, 3.53) more likely to have CMD as compared to those women who didn’t report any act of physical, sexual, or psychological violence or any combination of the three by an intimate partner during the current pregnancy.

The odds of developing CMD were three times (AOR = 3.10, 95% CI 1.65, 5.84) higher among nulliparous women as compared with a multiparous woman. Pregnant women who had been in the first trimester (AOR = 2.22, 95% CI 1.01, 4.93) and third trimester (AOR = 1.74, 95% CI 1.31, 2.31) were 2.2 times and 1.74 times more likely to have CMD as compared to those pregnant on the second trimester, respectively. Women with a history of abortion are two times (AOR = 2.03, 95% CI 1.27, 3.24) more likely to experience antenatal CMD. Pregnant women who didn’t have an ANC follow-up for their current pregnancy were 1.43 times (AOR = 1.43, 95% CI 1.08, 1.89) more likely to develop CMD as compared to those who had an ANC visit (Table 3).

**TABLE 3:** Results of bivariate and multivariate analysis for factors associated with antenatal common mental disorders.

		Common Mental Disorder			
Variables		Yes, *n* (%)	No, *n* (%)	Crude OR (95% CI)	Adjusted OR (95% CI)
Age	<20	31 (33.0)	63 (67.0)	1.00	1.00
	20–35	251 (37.1)	425 (62.9)	1.20 (0.76–1.89)	1.21 (0.74–1.99)
	>35	99 (40.4)	146 (59.6)	1.38 (0.84–2.27)	1.20 (0.69–2.10)
Wealth index	First quantile	154 (39.7)	234 (60.3)	1.34 (0.96–1.87)	1.26 (0.88–1.81)
	Second quantile	144 (38.4)	231 (61.6)	1.27 (0.91–1.77)	1.27 (0.89–1.82)
	Third quantile	83 (32.9)	169 (67.1)	1.00	1.00
History of mental illness	Yes	14 (53.8)	12 (46.2)	1.98 (0.90–4.32)	1.63 (0.68–3.86)
	No	367 (37.1)	622 (62.9)	1.00	1.00
History of chronic medical illness	Yes	9 (39.1)	14 (60.9)	1.07 (0.46–2.49)	0.74 (0.28–1.91)
	No	372 (37.5)	620 (62.5)	1.00	1.00
Current use of substance	Yes	81 (51.3)	77 (48.7)	1.95 (1.39–2.75)	1.99 (1.37–2.88)***
	No	300 (35.0)	557 (65.0)	1.00	1.00
Intimate partner violence	Yes	244 (49.5)	249 (50.5)	2.75 (2.15–3.58)	2.67 (2.02–3.53)***
	No	137 (26.5)	385 (73.5)	1.00	1.00
Parity	Nulliparous	29 (55.8)	23 (44.2)	2.54 (1.42–4.52)	3.10 (1.65–5.84)***
	Multiparous	163 (33.2)	328 (66.8)	1.00	1.00
	Grand multiparous	189 (40.0)	283 (60.0)	1.34 (1.03–1.75)	1.21 (0.90–1.62)
Gestational age (in week)	First trimester	16 (51.6)	15 (48.4)	2.35 (1.12–4.84)	2.22 (1.01–4.93)*
	Second trimester	143 (31.4)	312 (68.6)	1.00	1.00
	Third trimester	222 (42.0)	307 (58.0)	1.58 (1.21–2.05)	1.74 (1.31–2.31)***
History of abortion	Yes	50 (51.0)	48 (49.0)	1.84 (1.21–2.80)	2.03 (1.27–3.24)**
	No	331 (36.1)	586 (63.9)	1.00	1.00
ANC follow up	Yes	154 (31.6)	334 (68.4)	1.00	1.00
	No	227 (43.1)	300 (56.9)	1.64 (1.27–2.12)	1.43 (1.08–1.89)**
History of gynecological problem	Yes	36 (51.4)	34 (48.6)	1.84 (1.13–2.99)	1.69 (0.99–2.89)
	No	345 (36.5)	600 (63.5)	1.00	1.00
History of gynecological operation	Yes	23 (50.0)	23 (50.0)	1.71 (0.94–3.09)	1.57 (0.81–3.01)
	No	358 (36.9)	611 (63.1)	1.00	1.00

*P-value < 0.05; **P-value < 0.01; ***P-value < 0.001.

## Discussion

Almost two in five (37.5%) pregnant women had a common mental disorder in this study. In addition, current substance use, intimate partner violence, parity, gestational age, history of abortion, and lack of ANC attendance increased the odds of CMD.

The prevalence of 37.5% (95% CI: 34.5, 40.5) for common mental disorder among pregnant women in this study is consistent with other findings found in Bale, Ethiopia (35.8%) (19), Tanzania (39.5%) (43), South Africa (39%) (44), and Vietnam (37.4%) (45).

On the other hand, this result was higher than studies conducted in various regions of Ethiopia and other countries; Butajira (12%) (46), Gondar (23%) (47), and Addis Ababa (24.94%) (48), Brazil (20.2%) (49), and Nigeria (24.5%) (49). Likewise, in one community-based cross-sectional survey done in 20 sites across four countries, the prevalence of CMD was 21% in Vietnam, 33% in Ethiopia, 30% in India, and 30% in Peru (50). This difference might be due to the high magnitude of substance use, particularly Khat, in the current study setting, which significantly contributed to the development of mental illness (51). Additionally, poor utilization of ANC follow-up in the study setting might contribute to high maternal CMD.

The result is, however, lower than studies done in Jamaica (56%) (52), Turkish women (47.6%) (53), Nicaragua (57%) (54), Pakistan (49%) (55), and India (65%) (56). These variations might be due to disparities in measurement tools, and cut-off points used, Jamaican study used Zung self-rating depression scale (SDS > 50), Edinburgh postnatal depression scale (EDS) and the state and trait anxiety scale (STAI) were used in Turkey, India, and Nicaragua studies while Anxiety and Depression Scale (HADS, 11–21) were used in Pakistan. Conjointly, socio-cultural and economic variations, and geographical settings might also explain the observed difference.

In congruent with population-based cohort studies conducted in Ethiopia (57), India (58), Jordan (59), and Netherlands (60), pregnant women who use any type of substance were more likely to have CMD compared to those women who did not use any substance in the past 3 months. Mental health and substance use issues have a bidirectional cause-and-effect relationship. A pregnant woman using a substance often has complex social and mental health problems (51, 61). On the other hand, antenatal anxiety and depression could have a significant role in the causal mechanisms of substance use and prenatal stress, leading individuals to poor stress management and behavioral problems, both of which increase the risk of substance use disorder (62, 63). Similarly, a recent cross-sectional study found pregnant women with antenatal distress were more likely to chew Khat, as the women benefited from stimulant effects of Khat, including elation and alleviation of their symptoms (64).

Pregnant women, who reported intimate partner violence, were more likely to have CMD than those who did not report any acts of physical, sexual, or psychological violence or any combination of the three by an intimate partner during the current pregnancy. A similar finding was reported from the studies conducted in other areas of Ethiopia, Bale (19) and Butajira (65), Tanzania (66), and Vietnam (67). This might be because IPV can directly affect antenatal mental illnesses (68). First, having experienced partner violence, pregnant women may develop a stress response profile which heightens the neuroendocrine responses (69, 70). The collective effect of these responses can lead to mental illnesses. Secondly, women who had perinatal mental illness are more likely to be exposed to IPV during pregnancy than those who do not (71, 72). Finally, psychiatric disorders and IPV can happen along with structural stressors, such as low socioeconomic status or poor social support (73).

Nulliparous women were more likely to have CMD as compared to multiparous women. This finding was in agreement with studies done in Ethiopia (74), Pakistan (75), Vietnam (76), and Finland (77). This might be because primigravida women do not have any previous delivery experience, may have uncertainty and fear of childbirth, and they don’t have the knowledge and skills to take care of a child, which may heighten the incidence of depression and increase vulnerability to other psychological morbidities. There have been contradictory findings in studies as some have found multiparous women are at increased risk of developing antenatal common mental illnesses (59, 78, 79), while others did not find a strong association between parity and perinatal mental disorder (80, 81).

Another factor associated with antenatal CMD is gestational age. Pregnant women were more likely to experience CMD during the first and third trimester compared to those in their second trimester. Previous longitudinal studies conducted in England (82), China (83), and Lithuania (84) found a similar trend. Thus, the increased prevalence of CMD in the third trimester might be linked with the proximity of giving birth. Additionally, these results might be induced by hormonal changes in the first trimester (85).

Furthermore, in our study, women with past pregnancy complications or abortion had a higher odds of antenatal CMD. This is similar to the findings from various other studies (86–88). This might be because women who had a previous abortion or pregnancy complications may develop different psychosocial problems, worrying about possible complications in the index pregnancy. This may induce fear, uncertainty, worry, and anxiety, further enhancing the risk of depression during the subsequent pregnancies (89, 90).

Consistent with previous studies conducted in Gondar (47) and Debre Markos (74), Ethiopia, pregnant women who did not have ANC follow-up for their current pregnancy were more likely to have CMDs than those who had an ANC visit. The most likely explanation for this association is that antenatal clinic attendance may construct maternal self-esteem and resiliency, increase chance to obtain information about pregnancy preparedness and minimize risk factors, and increase knowledge about complications during pregnancy, including mental illnesses (91, 92).

### Limitations of the Study

This study has some limitations that should be kept in mind when interpreting the results. SRQ-20 is a screening but not diagnostic tool. Beside, this report did not evaluate biological determinants of common mental disorders, such as thyroid dysfunction and immunity. Additionally, the study did not differentiate women with new-onset mental disorders from those with a pre-existing conditions. Lastly, since we recruited multiple data collectors, there may be interviewer bias.

## Conclusion

Pregnancy is a time of increased susceptibility for the occurrence of common mental disorder. Almost two in every five pregnant women suffered from antenatal CMD in the study area. Currently, using a substance in early and late pregnancy, experiencing intimate partner violence, null parity, having a history of abortion or pregnancy complications, and no ANC follow-up were significantly associated with antenatal CMD. Administration of regular screening programs for maternal mental health conditions in rural, low-income communities, integrating into primary health care settings is imperative to reduce the risk. This study also underscores the importance of routine maternal mental health assessment and intervention when pregnant women present to maternity services. Pregnant women in this setting should be informed that substance use in pregnancy should be avoided or reduced.

## Data Availability Statement

The original contributions presented in the study are included in the article/supplementary material, further inquiries can be directed to the corresponding author/s.

## Ethics Statement

The studies involving human participants were reviewed and approved by the Haramaya University, College of Health and Medical Sciences, Institutional Health Research Ethics Review Committee (IHRERC). The patients/participants provided their written informed consent to participate in this study.

## Author Contributions

All authors contributed to the conception of the study, organized the data collection process, equally contributed to data analysis, drafting or revising the article, gave final approval of the version to be published, and agreed to be accountable for all aspects of the work.

## Conflict of Interest

The authors declare that the research was conducted in the absence of any commercial or financial relationships that could be construed as a potential conflict of interest.

## Publisher’s Note

All claims expressed in this article are solely those of the authors and do not necessarily represent those of their affiliated organizations, or those of the publisher, the editors and the reviewers. Any product that may be evaluated in this article, or claim that may be made by its manufacturer, is not guaranteed or endorsed by the publisher.

## Abbreviations

ANC: antenatal care
AOR: adjusted odds ratio
ASSIST: Alcohol, Smoking and Substance Involvement Screening Test
CI: confidence interval
CMD: common mental disorder
COR: crude odds ratio
GTP: Growth and Transformation Plan
HDSS: health and demographic surveillance sites
IPV: intimate partner violence
MhGAP: Mental Health Gap Action Program
ODK: open data kit
SDG: sustainable development goals
SRQ-20: Self-Reporting Questionnaire
VIF: variance inflation factor
YLD: years lived with disability.
